# Phytoplasma infection of a tropical root crop triggers bottom-up cascades by favoring generalist over specialist herbivores

**DOI:** 10.1371/journal.pone.0182766

**Published:** 2017-08-16

**Authors:** Kris A. G. Wyckhuys, Ignazio Graziosi, Dharani Dhar Burra, Abigail Jan Walter

**Affiliations:** 1 International Center for Tropical Agriculture (CIAT) Asia Regional Office, Hanoi, Vietnam; 2 University of Kentucky, Lexington, Kentucky, United States of America; 3 Swedish University of Agricultural Sciences SLU, Alnarp, Sweden; Institut Sophia Agrobiotech, FRANCE

## Abstract

Global interest on plant-microbe-insect interactions is rapidly growing, revealing the multiple ways in which microorganisms mediate plant-herbivore interactions. Phytopathogens regularly alter whole repertoires of plant phenotypic traits, and bring about shifts in key chemical or morphological characteristics of plant hosts. Pathogens can also cause cascading effects on higher trophic levels, and eventually shape entire plant-associated arthropod communities. We tested the hypothesis that a *Candidatus* Phytoplasma causing cassava witches’ broom (CWB) on cassava (*Manihot esculenta* Grantz) is altering species composition of invasive herbivores and their associated parasitic hymenopterans. We conducted observational studies in cassava fields in eastern Cambodia to assess the effect of CWB infection on abundance of specialist and generalist mealybugs (Homoptera: Pseudococcidae), and associated primary and hyper-parasitoid species. CWB infection positively affects overall mealybug abundance and species richness at a plant- and field-level, and disproportionately favors a generalist mealybug over a specialist feeder. CWB phytoplasma infection led to increased parasitoid richness and diversity, with richness of ‘comparative’ specialist taxa being the most significantly affected. Parasitism rate did not differ among infected and uninfected plants, and mealybug host suppression was not impacted. CWB phytoplasma modifies host plant quality for sap-feeding homopterans, differentially affects success rates of two invasive species, and generates niche opportunities for higher trophic orders. By doing so, a *Candidatus* phytoplasma affects broader food web structure and functioning, and assumes the role of an ecosystem engineer. Our work unveils key facets of phytoplasma ecology, and sheds light upon complex multi-trophic interactions mediated by an emerging phytopathogen. These findings have further implications for invasion ecology and management.

## Introduction

In their natural environment, plants face concurrent and successive exposure to a variety of attackers, including insect herbivores, viruses, bacteria and fungi [1]. While single plant-antagonist combinations have been fairly well described, the dynamics of plant-herbivore interactions under multiple attack have only recently caught scientists’ attention [2,3]. Nevertheless, these plant-microbe-insect (PMI) interactions can have far-reaching impacts at multiple levels of biological organization, and may shape the composition and function of entire trophic communities [4]. Pathogen-mediated interactions should therefore preferably be assessed within the community context and examined through the lens of multi-trophic ecology [3,5,6].

Co-occurring antagonists can differentially affect defense signaling pathways and subsequently shape a plant’s volatile emissions or modify colonization and foraging patterns by plant-associated arthropods [2,7,8]. In addition to affecting herbivores, systemically-induced plant defenses and semiochemical release can also have major implications for higher trophic levels [9]. Furthermore, plant-microbe-insect interactions can affect the overall plant phenotype and alter the chemical, morphological and physiological traits of plants [10,11]. Pathogen-infected plants can also have vastly different color, architecture or micro-climate conditions [12], any of which could affect plant-herbivore interactions. Herbivore morphology, life history, fitness, and behavior can be impacted by plant quality, with cascading effects on natural enemies such as parasitoids and predators [13,14,15,16], thus affecting the composition, structure, and function of entire arthropod communities [3]. Lastly, pathogen-mediated effects tend to be highly species-specific and context-dependent, and can vary greatly among herbivores within the same feeding guild or family [17].

Although a rapidly growing body of literature covers the many intricate ways in which viral pathogens modulate host-vector interactions, the effect of other classes of plant pathogens is less well studied [18,19]. Examples from certain pathosystems show that bacterial and fungal pathogens often have subtle indirect impacts on herbivores and higher trophic levels. For example, mildew-infected plants are less attractive to the braconid parasitoid *Cotesia glomerata*, and pierid caterpillars on those plants have lower levels of parasitisation [20]. Also, citrus trees infected by the bacterium, *Candidatus* Liberibacter asiaticus have altered volatile blends and plant nutrient profiles that subsequently modify host choice, mate selection and movement patterns of the psyllid vector *Diaphorina citri* [21,22]. At present, it is widely accepted that phytopathogenic fungi or bacteria modulate populations of their vectors as much as non-vector herbivores, but the effect at higher trophic levels is yet to be fully understood [6].

One group of vector-borne bacterial pathogens that has received virtually no attention from a community ecology perspective are *Candidatus* Phytoplasma spp. [3]. Phytoplasma are phloem-limited bacteria that modify plant hormone balance and cause dramatic alterations in plant morphology, including extensive leaf proliferation, and creation of pseudo-flowers or witches’ broom symptoms [23]. These distinct plant morphologies may create niche opportunities for plant-associated species, alter the foraging success of natural enemies, or create enemy-free space for herbivores [24]. Considered ‘expert manipulators’ of sieve elements, phytoplasma cause marked shifts in hormone balances, energy flows and phloem content [25,26], and could thus affect the nutritional ecology of phloem-feeders such as aphids, scales or mealybugs [27]. Despite the association of phytoplasma with several economically-important diseases, these pathosystems have remained critically under-researched [28]. One particular knowledge gap concerns the extent to which individual herbivore species or arthropod communities are influenced by phytoplasma-mediated alterations in plant phenotype [23,29].

Much of the past work on cross-kingdom interactions has not deliberately focused on non-native versus endemic organisms, or contrasted the response of antagonists of varying levels of dietary specialization. Nevertheless, non-native phytopathogens can have dramatic effects on food webs, and can facilitate the spread of invading arthropods [30,31]. Also, pathogen-mediated changes in host plant quality can differentially affect development and performance of specialist and generalist herbivores. Through the ‘tri-trophic interactions hypothesis’ (TTI), specialists are predicted to be more dominant than generalists and experience higher evolutionary success on low-quality plants, with plant quality and palatability greatly increased by pathogen co-infection [32]. To our knowledge, no research has been conducted on the extent to which a specific phytopathogen shapes relative success ratios of invasive herbivores of differing dietary specialization, and their associated trophic communities.

We tested the TTI hypothesis through observational studies in a tropical agro-ecosystem, using the unique case of a semi-perennial host plant that is concurrently attacked by a non-native systemic plant pathogen and two non-native phloem-feeders. More specifically, we assessed performance of two invasive mealybugs in cassava (*Manihot esculenta*); a tropical root crop extensively grown by smallholder farmers throughout Southeast Asia. In recent years, a number of non-native mealybug (Hemiptera: Pseudococcidae) species have colonized Asia’s cassava fields [33], including the specialist *Phenacoccus manihoti* Matile-Ferrero, a Neotropical parthenogenetic herbivore (9 recorded host species); and the generalist *Paracoccus marginatus* Williams & Granara de Willink, a Nearctic sexual herbivore (133 host genera). Invasion history is fairly similar for both species, with respective colonization of Asian cassava presumably initiated around 2008 and 2010 [34]. Biological control of both species has been attempted, with the encyrtids *Anagyrus lopezi* De Santis (for *P*. *manihoti*, released in 2009), and *Acerophagus papayae* Noyes & Schauff released against *P*. *manihoti* (2009) and *P*. *marginatus* (2010) respectively. Furthermore, cassava fields have been invaded by cassava witches broom (CWB) disease, an emerging pathogen associated with multiple strains of *Candidatus* Phytoplasma [33,35]. CWB-affected plants exhibit distinctive leaf discoloration, extensive proliferation of leaves and stems, and stunted growth.

We assessed the effect of CWB-infection on the abundance and diversity of mealybugs and their associated parasitoid communities. We focused on 3 research questions: (1) Does CWB infection affect the relative abundance of specialist versus generalist herbivores within the same feeding guild and insect family? (2) Does CWB phytoplasma infection alter the abundance and composition of parasitoid and hyperparasitoid assemblages, this affecting mealybug biological control? (3) Does phytoplasma infection create new habitat and species-specific niche opportunities for invasive species? We reveal how phytoplasma infection is shaping entire host x parasitoid communities in cassava fields, add understanding of phytoplasma-mediated ecological processes, and inform management interventions that benefit insect biological control in a different context.

## Materials and methods

We surveyed farmer-managed cassava fields located in Kracheh province (eastern Cambodia) over the course of two months (January-February) during the 2016 dry season. Selection of fields was done in close collaboration with officials from the Kracheh Provincial Department of Agriculture (PDA), Ministry of Agriculture, Forestry and Fisheries (MAFF) of the Royal Government of Cambodia, and with consent from individual farmers. Observational studies were done in two fields in each of four sites, based on local availability of fields with differing levels of infection by cassava witches’ broom disease (CWB). Geographical coordinates (Lat/Long) of the districts and villages where fields were selected as follows: Chetborey, Changchrong (12.58275°, 106.07681°), Chetborey, Sambuk (12.64369°, 106.06290°), Prakprosab, Bang Liegh (12.35089°, 105.98528°), Snoul (12.02920°, 106.40232°). Within each site, we selected one field with high (>20%) and one field with low (0–5%) incidence of CWB, as determined by assessing the presence of plants with typical symptoms of CWB infection (i.e., stunting, leaf yellowing, leaf and petiole proliferation). Incidence rates refer to the percentage plants within a given field with above disease symptoms, and incidence categories (i.e., high, low) were defined based upon prevailing CWB infection rates in Kracheh province. Those symptoms are typical for infestation by CWB phytoplasma and cannot be ascribed to any other disease, infection or herbivore attack [33]. Though phytoplasma regularly exhibit an uneven distribution in infected plant tissue [36], CWB phytoplasma was positively detected through PCR from 70–100% of symptomatic tissue collected from Laos and Vietnam [33]. Sites were at least 7 km apart, with individual field plots located at minimum 2 km from each other. Fields measured 1 to 4 ha in size, close to maturity (8–9 months old), planted with a locally common variety and exclusively managed by growers using the same practices.

Within each field, we first assessed local incidence and plant-level population pressure of different mealybug species using an established transect-based monitoring protocol [33]. In brief, we recorded mealybug population levels and CWB-infection status on a total of 50 plants in each field, positioned along five transects randomly distributed throughout the field. In addition to field-level monitoring, we randomly sampled four mealybug-infested plants without CWB symptoms from each field, and an additional four mealybug-infested CWB-symptomatic plants from plots with high CWB incidence (N = 64; total number of samples). To investigate whether CWB infection altered mealybug and parasitoid populations at a field level, we opted to contrast herbivore and parasitoid populations between symptomatic and asymptomatic plants only in setting with high CWB incidence. We thus obtained mealybug-infested plant samples in three categories, based upon field- or plant-level infection with CWB: CWB_NN (asymptomatic plants in ‘CWB-free’ plots), CWB_YN (asymptomatic plants, in CWB-affected plots) and CWB_YY (symptomatic plants, in CWB-affected plots). Plants were chosen based upon the presence of mealybugs on leaves and stems or on deformed leaves (so-called bunchy tops). We sampled each plant by cutting the apical part at 18 cm from the growth tip, and placing into a 27 x 32 x 13cm paper bag. Bags were subsequently sealed, placed in a cooler box and transported to a field laboratory for further processing. All field collections were conducted between 8:30 and 10:00 am.

Upon arrival in the laboratory, we carefully removed plants from the bags, and counted and identified all mealybug individuals. Other arthropods occurring on the plant (e.g., spiders, lacewings, ladybeetles) were initially identified based on morpho-type, and then stored in 95% ethanol for further identification. Cassava plant tips were then transferred individually to 20 x 14 x 14 cm transparent polyvinyl chloride (PVC) containers, and closed with fine cotton fabric mesh to prevent escape of arthropods. We checked containers daily for emergence of parasitoids or hyperparasitoids over a period of 14 days, Emerging insects were removed from containers using an aspirator, photographed, identified to morpho-type and stored in ethanol for subsequent species-level identification. Primary parasitoids are generally attacked by hyperparasitoids or mummy parasitoids, but no distinction was made between both guilds. As our experimental protocol did not allow distinguishing the exact herbivore or (primary) parasitoid host of particular hyperparasitoids, we used the parasitoid / host ratio as proxy for parasitism rates. Host range and ecological role of each of the different parasitoid species was queried in the Universal Chalcidoidea Database [37] and Taxapad [38]. These databases were equally used to indicate whether particular species either acted as primary parasitoids, hyperparasitoids (or both). Data were used to calculate a Simpsons’ Index for each plant sample, as a measure of parasitoid species richness and diversity. Differences in parasitoid assemblages for each sample were characterized by non-metric multidimensional scaling (NMDS) ordination using *metaMDS* in the R package Vegan [39], and the resulting ordination plot was visualized using the R package ggplot2 [40]. A Bray-Curtis dissimilarity matrix was calculated using *vegdist* in package Vegan, and permutational multivariate analysis of variance using distance matrices *(PERMANOVA)* analysis was performed using Adonis in the R package Vegan. In the model, the influence of plant-level CWB infection status, i.e. CWB_NN (asymptomatic plants in CWB-free plots), CWB_YN (asymptomatic plants, in CWB-affected plots) and CWB_YY (symptomatic plants, in CWB-affected plots), on the dissimilarity matrix was calculated, in order to identify significant differences in parasitoid assemblage abundances between different plant-level CWB infection statuses. Furthermore *betadisper* in the R package Vegan was used to test homogeneity of variance assumption of the PERMANOVA procedure.

In all analyses, we considered three different CWB infection statuses: CWB_NN (asymptomatic plants in CWB-free plots), CWB_YN (asymptomatic plants in CWB-affected plots), and CWB_YY (symptomatic plants in CWB-affected plots). We tested the effect of plant-level and field-level CWB infection on the relative abundance and sex ratio of both herbivores and parasitoids using multivariate analysis of variance (MANOVA). We employed non-parametric tests (e.g., Kruskal-Wallis) since data were not normal. Parasitism rates, parasitoid richness, and diversity were also compared between different sites. Data were tested for normality and homoscedasticity, and subsequently square root-transformed. Statistical analyses were performed using SPSS [41].

## Results

### Herbivore abundance and species composition

**Fig 1:**
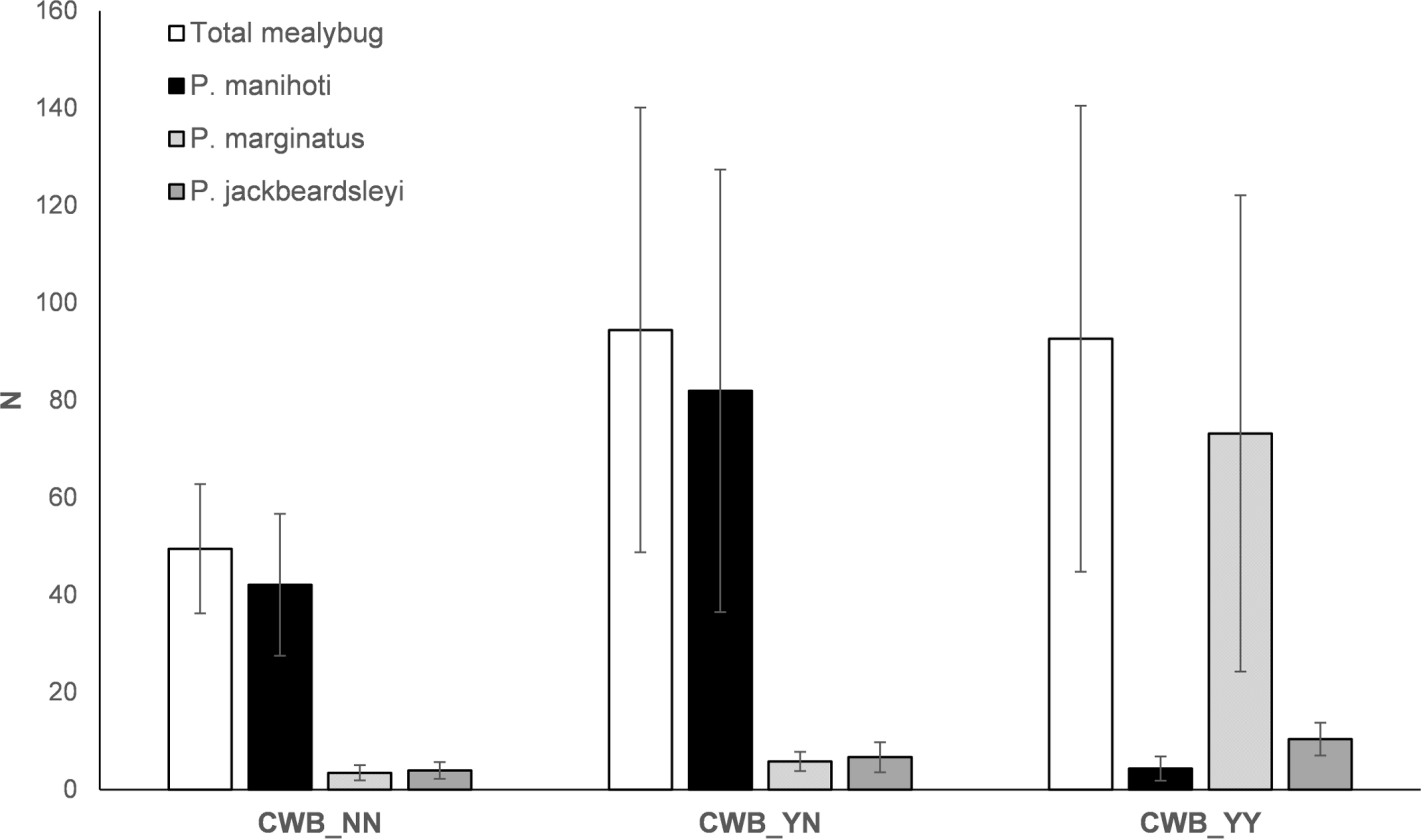
Plant-level abundance of the three dominant invasive mealybug species, under contexts of different plant- and field-level infection with cassava witches’ broom (CWB) disease. Abundance ratios are presented for CWB_NN (uninfected plants, in CWB-free plots), CWB_YN (uninfected plants, in CWB-affected plots), CWB_YY (infected plants, in CWB-affected plots). Asterisks in the graph indicate statistically significant differences (ANOVA, Tukey HSD posthoc, α = 0.05).

Over the course of the experiment, a total of 3,785 mealybugs were recorded on cassava plants. The mealybug community was primarily composed of *P*. *manihoti* (54.2%) and *P*. *marginatus* (34.8%), while *P*. *jackbeardsleyi* represented 8.9% and *Ferrisia virgata* 2.0% of the total species complex (Table 1; S1 Table). Overall mealybug abundance varied greatly between research sites and fields (Fig 1). The specialist *P*. *manihoti* was the most abundant species on CWB-asymptomatic plants (CWB_NN and CWB_YN), while abundance of the generalist mealybug *Pa*. *marginatus* was significantly higher compared to the other taxa on CWB-symptomatic plants (Fig 1). Total mealybug abundance was significantly affected by site (ANOVA, F_3,36_ = 3.748, p = 0.001), and marginally significant for a site x CWB infection status interaction (F_6,36_ = 2.155, p = 0.071). For *P*. *manihoti*, significant effects were recorded for site (F_3,16_ = 5.127, p = 0.011) and CWB infection status (F_2,16_ = 6.932, p = 0.007). For *Pa*. *marginatus*, significant effects were noted for CWB infection status (F_2,16_ = 6.113, p = 0.011), and a site x CWB infection status interaction (F_2,16_ = 4.701, p = 0.025).

**Table 1:** Principal herbivore, parasitoid and hyperparasitoid species recorded during early 2015 from mealybug-infected cassava plants in Kracheh province, eastern Cambodia.

Feeding guild	Family / species	Host range (n)^a^	Main hosts	Hyper- parasitoid
***Herbivore***				
**Homoptera, Pseudococcidae**				
	1. *Phenacoccus manihoti*	9	Cassava	**-**^b^
	2. *Paracoccus marginatus*	133	papaya, cassava, mulberry	**-**
	3. *Pseudococcus jackbeardsleyi*	98	mango, banana, cacao, tea, cassava	**-**
***Primary parasitoid / hyper- or mummy parasitoid***				
**Hymenoptera, Encyrtidae**				
	1. *Anagyrus lopezi*	4	*P*. *manihoti*	No
	2. *Acerophagus papayae*	1	*P*. *marginatus*	No
	3. *Prochiloneurus pulchellus*	30	*P*. *marginatus*, *P*. *manihoti*	Yes
	4. *Anagyrus* sp.	4	*P*. *marginatus*	No
	5. *Pseudoleptomastix* sp.	1	*P*. *marginatus*	No
	6. *Aenasius advena*	14	*P*. *jackbeardsleyi*, *P*. *manihoti*, *Ferrisia virgate*	No
**Hymenoptera, Eriaporidae**				
	7. *Promuscidae unfasciativentris*	18	*F*. *virgata*	Yes
**Hymenoptera, Aphelinidae**				
	8. *Marietta leopardina*	**-**	**-**	Yes

For each of the different trophic groups and insect families, primary species are listed and baseline information is provided on their associated host plants, mealybug hosts, and eventual status as hyperparasitoid (or mummy parasitoid).

^a^ Host range data cover plant hosts for herbivores and mealybug hosts for primary parasitoids, as distilled from Ben-Dov et al. (2016), Noyes (2016) and Yu et al. (2012). Primary host records were also obtained from the above sources.

^b^ Not applicable

CWB-infection status significantly increased mealybug richness (Kruskal Wallis, *Χ* = 6.172, p = 0.046), as well as mealybug diversity (*Χ* = 7.926, p = 0.019). On infected plants within CWB-affected plots, mealybug diversity was three times higher than in un-affected plants in disease-free fields (i.e., species diversity 0.09 ± 0.16 vs. 0.28 ± 0.26).

### Parasitoid and hyperparasitoid communities

A total of 888 parasitoids and hyperparasitoids were reared from field-collected mealybugs (Table 1). The parasitoid community was composed of 11 different (morpho-)species, with *A*. *lopezi*, *A*. *papayae* and *Prochiloneurus* sp. representing 46.4%, 13.4% and 12.5% respectively of the entire community. Overall parasitoid abundance was significantly affected by research site (F_3,29_ = 3.962, p = 0.018), and by a site x CWB infestation status interaction (F_6,29_ = 2.533, p = 0.043) (Table 2). For abundance of comparative specialists, marginally significant effects were noted for site (F_3,25_ = 2.781, p = 0.062), and a site x infection status interaction (F_6,25_ = 2.956, p = 0.025). Hyper-parasitoid abundance was not significantly affected by site or CWB infection status. For *A*. *lopezi*, (marginally) significant effects on abundance were noted for site (F_3,19_ = 3.963, p = 0.024), CWB infection status (F_2,19_ = 2.268, p = 0.131) and a site x infection status interaction (F_4,19_ = 4.534, p = 0.010); for *A*. *papayae*, CWB infection status significantly affected parasitoid abundance (F_2,6_ = 7.695, p = 0.022); while for *Prochiloneurus* sp. and *Pseudoleptomastix* sp. no significant effects of site nor CWB infection status were detected.

**Table 2:** Aggregate and species-specific abundance measures (average ± SD) for the most abundant parasitoid and hyperparasitoid species, as reared from mealybug-infested plants.

Abundance measure	Pathogen infection status			Test statistic
	CWB_NN	CWB_YN	CWB_YY	
**Total parasitoid abundance**	19.43 ± 35.10a	13.75 ± 16.31a	22.31 ± 37.62a	F_2,38_ = 0.59, *p* = 0.56
**Specialist abundance**	13.81 ± 22.41a	10.62 ± 12.74a	14.68 ± 25.29a	F_2,34_ = 0.01, p = 0.99
**Hyper-parasitoid abundance**	4.37 ± 11.80a	1.93 ± 2.59a	2.62 ± 6.88a	F_2,18_ = 1.16, p = 0.33
***A*. *lopezi***	13.31 ± 22.60a	9.93 ± 12.25a	2.50 ± 5.38a	F_2,26_ = 1.11, *p* = 0.34
***A*. *papayae***	0.12 ± 0.50a	0.19 ± 0.40a	7.12 ± 18.29b	**F**_**2,6**_ **= 7.69, *p* = 0.02**
***Prochiloneurus pulchellus***	2.87 ± 6.24a	1.62 ± 2.22a	2.43 ± 6.18a	F_2,17_ = 2.18, *p* = 0.14
***Pseudoleptomastix* sp.**	0.12 ± 0.50a	0.37 ±± 0.88a	4.69 ± 7.92a	F_2,11_ = 1.81, *p* = 0.21

Parasitoid abundance levels are listed for CWB_NN (asymptomatic plants, in CWB-free plots), CWB_YN (asymptomatic plants, in CWB-affected plots), CWB_YY (symptomatic plants, in CWB-affected plots). Values within the same row followed by identical letters are not statistically significant (ANOVA, honest significant difference Tukey posthoc, α = 0.05).

**Fig 2:**
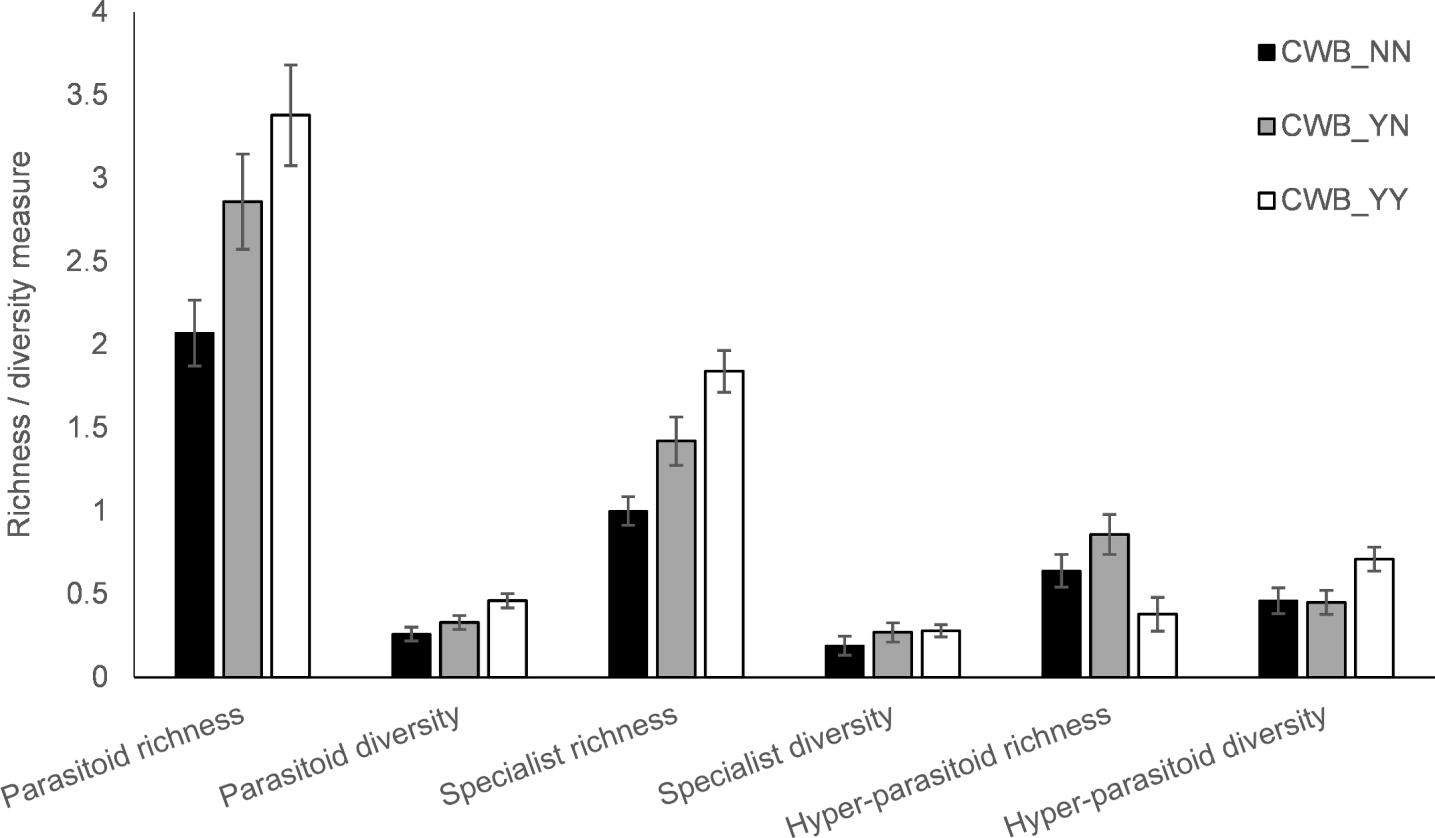
Species richness and diversity indices for total parasitoids, comparative specialists and hyper-parasitoids, as recorded under contexts of varying plant- and field-level infection with cassava witches’ broom (CWB) disease. Richness and diversity indices are presented for CWB_NN (uninfected plants, in CWB-free plots), CWB_YN (uninfected plants, in CWB-affected plots), CWB_YY (infected plants, in CWB-affected plots). Asterisks indicate level of significance, with ** highly significant at p<0.01, and * marginally significant at p<0.1 (Mann-Whitney U).

**Fig 3:**
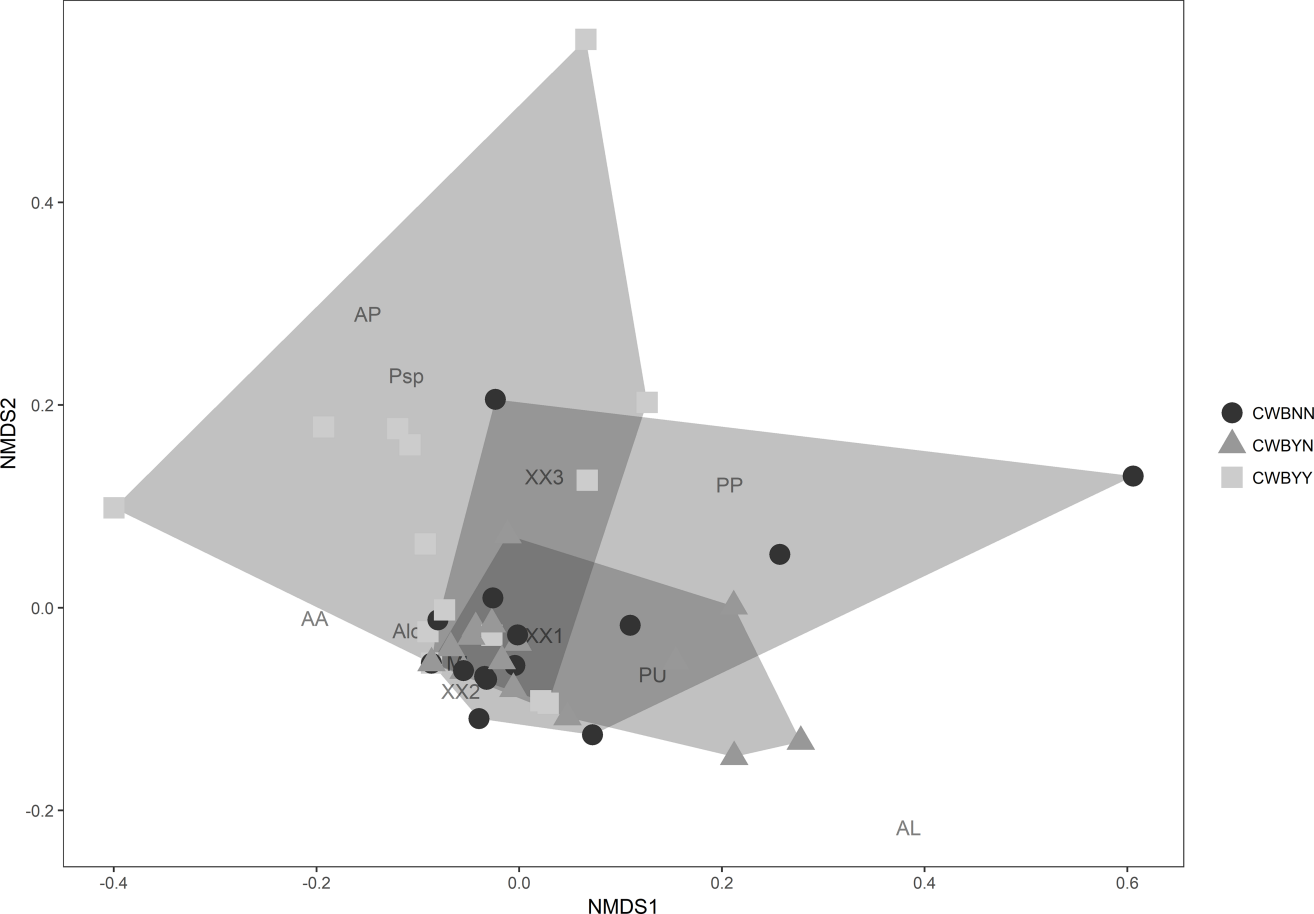
Non-metric multidimensional scaling (NMDS) ordination of different plant-level CWB infection status based on parasitoid assemblages, specifically on species-level and morphotype abundance levels. Clustering within the NMDS ordination space shows significantly different (PERMANOVA, F (2, 45) = 2.34, p = 0.02) parasitoid assemblages on phytoplasma-infected plants (CWB_YY; grey squares), on un-infected plants in CWB-affected plots (CWB_YN; grey triangles) and uninfected fields (CWB_NN; black circles). The dominant parasitoid species within specific assemblages is depicted, as such: AL (*A*. *lopezi*), AP (*A*. *papayae*), PP (*Prochiloneurus* sp.), ALo (*Anagyrus* sp.), PU (*P*. *unfasciativentris*), ML (*M*. *leopardina*), AA (*A*. *advena*), Psp (*Pseudoleptomastix* sp.) and 3 unidentified species (XX1, 2, 3).

Field- and plant-level pathogen-infection significantly affected richness of specialist parasitoids (Kruskal Wallis *Χ* = 6.98, p = 0.03), and had marginally significant effects on total parasitoid richness and diversity (Fig 2). Significantly distinct parasitoid complexes *(PERMANOVA*, *F*_*(2*, *45)*_ *= 2*.*34*, *p = 0*.*02)*, differentiated by plant-level CWB infection status were observed through NMDS (Fig 3).

### Parasitism rate, and diversity x function relationship

**Fig 4:**
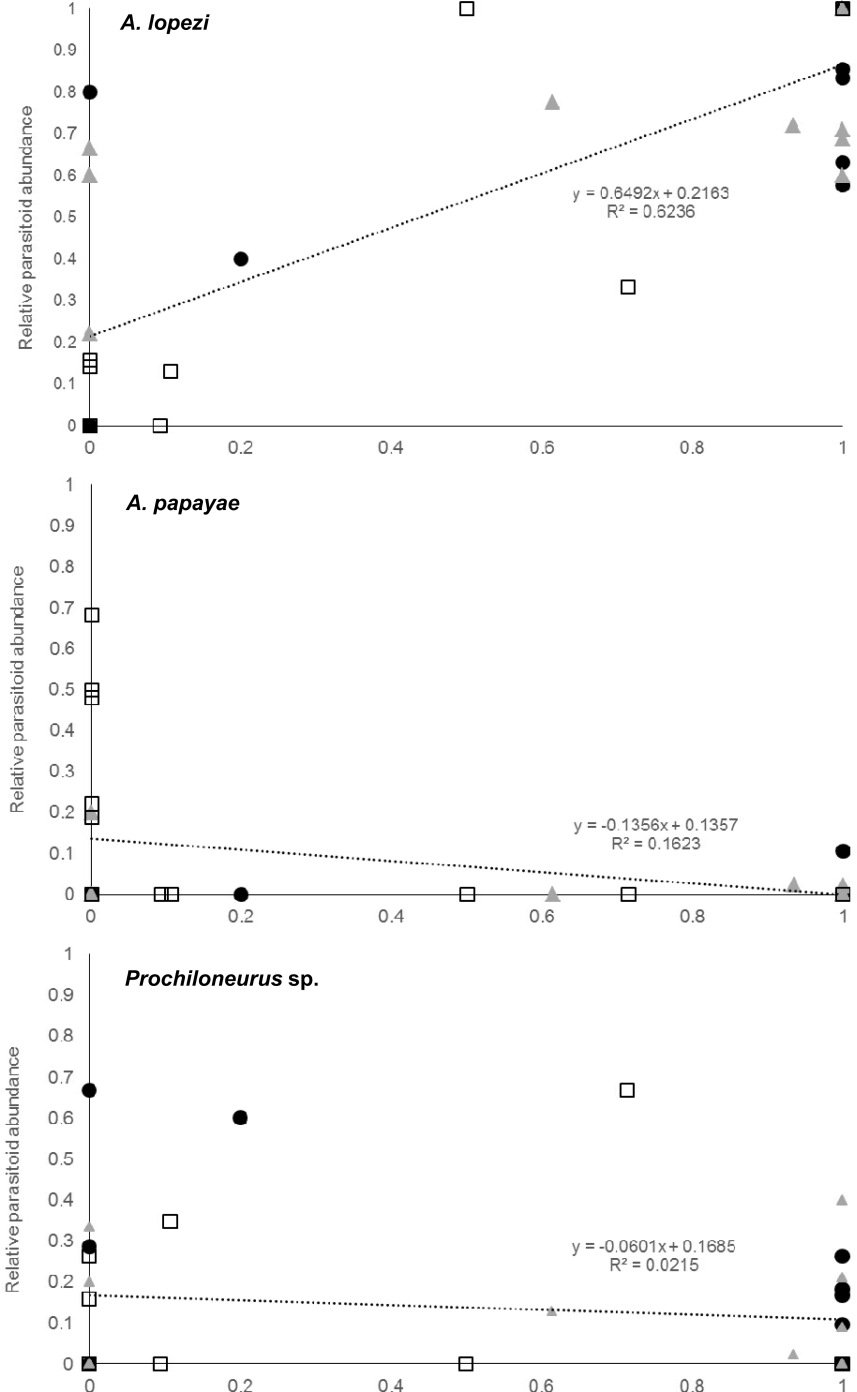
Relative abundance of three parasitoid species, on cassava plants with mealybug communities of variable species composition. Mealybug species composition is represented by the relative proportion of *P*. *manihoti* versus *Pa*. *marginatus*. Symbols reflect plant- and field-level CWB infection status.

Parasitism rates varied greatly according to field, site and CWB-infection. Total parasitism did not vary significantly depending sites or CWB-infection status. For *A*. *lopezi* parasitism levels, no differences were found between sites or CWB infection contexts, but *A*. *papayae* parasitism was significantly affected by site (F_3,14_ = 5.119, p = 0.013), CWB infection (F_2,14_ = 6.317, p = 0.011) and a site x infection status interaction (F_1,14_ = 9.353, p = 0.009). Relative abundance of *A*. *lopezi* significantly increased in *P*. *manihoti*-dominated mealybug colonies (F_1,35_ = 7.423, p< 0.001; Fig 4), while relative abundance of *A*. *papayae* was significantly lower in *P*. *manihoti* colonies (F_1,35_ = 6.398, p = 0.016). CWB-infection status had a marginally-significant effect on *A*. *lopezi* sex ratio (F_2,26_ = 2.613, p = 0.092), with more male-biased sex ratios on CWB-infected plants.

**Fig 5:**
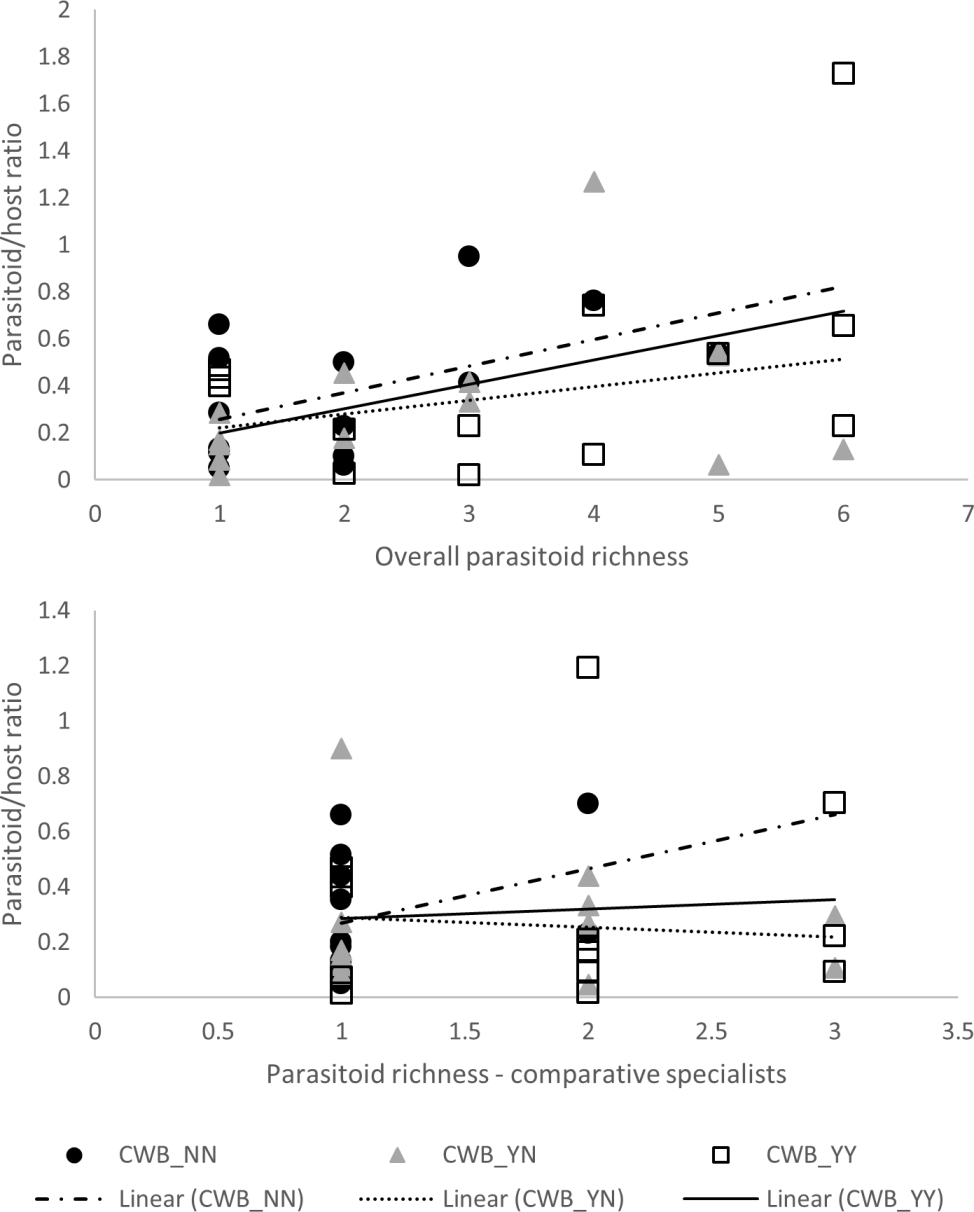
Diversity-function relationship, as represented by species richness of total parasitoids or comparative specialists versus parasitoid / host ratio (arcsin square root transformed). Regression curves are presented for different conditions of field- and plant-level disease incidence: CWB_NN (uninfected plants, in CWB-free plots), CWB_YN (uninfected plants, in CWB-affected plots), CWB_YY (infected plants, in CWB-affected plots). No significant interaction effects were observed for CWB infection status.

**Fig 6:**
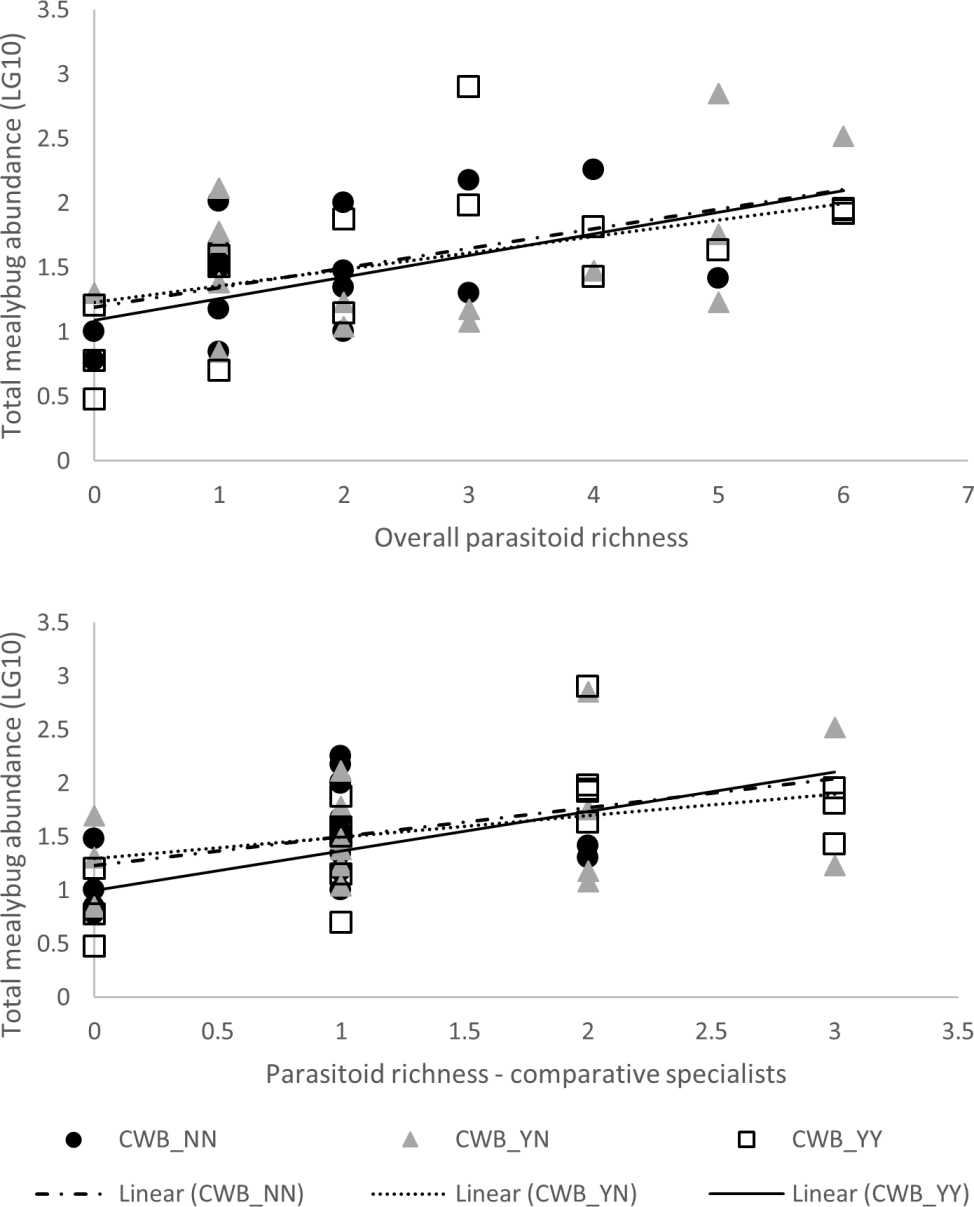
Diversity-function relationship, as represented by species richness of total parasitoids or comparative specialists versus total mealybug abundance (LG10 transformed). Regression curves are presented for different conditions of field- and plant-level disease incidence: CWB_NN (uninfected plants, in CWB-free plots), CWB_YN (uninfected plants, in CWB-affected plots), CWB_YY (infected plants, in CWB-affected plots). No significant interaction effects were observed for CWB infection status.

Total parasitism rates were consistently higher in settings with species-rich parasitoid communities, but species-rich parasitoid communities did not result in lower mealybug abundance (Figs 5 and 6). Highly significant regression patterns were recorded for total parasitoid or specialist parasitoid species richness and parasitism rate were observed (F_1,44_ = 15.304, p<0.001; F_1,44_ = 17.143, p<0.001). No interaction effects were recorded for CWB-infection status for either total parasitoid or specialist parasitoid richness (ANCOVA, interaction effect: F_7,32_ = 1.680, p = 0.149; F_4,38_ = 1.908, p = 0.129). Regression analysis revealed significant correlation between total parasitoid or specialist parasitoid species richness and total mealybug abundance (F_1,46_ = 17.450, p<0.001; F_1,46_ = 13.411, p = 0.001). Once again, no interaction effects were recorded for CWB-infection status for either variable (F_7,34_ = 0.651, p = 0.711; F_4,40_ = 1.420, p = 0.245).

## Discussion

Despite the flourishing research on cross-kingdom interactions, only scant information exists about the ways in which *Candidatus* Phytoplasma spp. affect herbivores or shape arthropod communities on plants. Recent work has revealed that other phytopathogens, e.g., viruses, fungi and bacteria, affect defense response pathways and induce changes in plant physiology, morphology and chemistry [10]. A number of pathogens are even termed ‘manipulative parasites’ for the extent to which they attract or discourage potential insect vectors or non-vector organisms [4,42]. Certain pathogens modulate behavior, fitness and population dynamics of individual arthropods and determine structure and function of entire trophic communities [18,43,44]. This study provides a first account of how a *Candidatus* phytoplasma triggers bottom-up cascades and affects complex herbivore x parasitoid x hyperparasitoid assemblages. Although CWB phytoplasma creates entirely new habitats for insect colonist through modified plant metabolites, defense chemicals, and plant architecture, it is challenging to fully capture the evolutionary and ecological forces that act among the various organisms in our study system. Nevertheless, we postulate a number of different hypotheses and highlight priority areas for follow-up research.

Biotrophic pathogens such as phytoplasma trigger salicylate-dependent defense pathways, but also ‘hijack’ a plant’s immune system through release of specific effector proteins [21,45]. Phloem-feeding herbivores such as mealybugs equally induce hormonal defenses, and regularly manipulate the hormonal response of a plant to their own advantage [46]. The unique case of CWB phytoplasma simultaneously facilitating generalist and inhibiting specialist phloem feeders, i.e., *Pa*. *marginatus* or *P*. *manihoti* respectively, supports the notion that plant-phytoplasma interactions change host quality for herbivores, with the outcome dependent upon species-specific plant-herbivore interactions [27].

Induction of plant defense mechanisms can also result in suppression or increased emission of specific volatile organic compounds (VOCs) [2]. Recent work shows that Phytoplasma spp. induce shifts in plant volatile emission patterns [47], and thus may possibly modulate herbivore or natural enemy foraging patterns [2,6,7,48]. In our study system, this could explain the altered patterns of mealybug occurrence or the higher incidence of parasitic wasps on CWB-affected plants. However, the relative importance of phytohormonal and volatile-mediated effects of CWB phytoplasma, as for many other pathogens, waits to be investigated [2].

Phytopathogens regularly modify plant traits and thus create different niche opportunities for herbivores. The increased plant branching and leafy morphologies of CWB-affected plants may differentially affect herbivore success or alter foraging patterns of natural enemies such as ladybeetles or lacewings [24,49]. Also, phytoplasma infection impacts phloem transport or leaf chemistry, and substantially increases levels of phagostimulatory sugars or starch [25,26]. These kinds of pathogen-mediated shifts in plant quality can impact palatability and attractiveness for herbivores. For example, heightened amino acid levels of fungus-infected birch leaves lead to increased size, weight and fitness in the aphid *Euceraphis betulae* [5]. Future research can investigate whether CWB-affected plants have increased amino acid or carbohydrate content, and thus provide disproportionate fitness benefits for generalist herbivores such as *Pa*. *marginatus*.

A plant’s secondary chemistry profile can have cascading effects on higher trophic levels, and lead to a so-called community phenotype [1,14,50,51]. More so, certain phytotoxins accumulate along trophic chains and cause ‘toxic environmental effects’ [52]. This is particularly relevant for cassava, a plant that employs extensive chemical defenses such as cyanogenic and flavonoid glycosides and hydroxycoumarins [53]. Quantitative variation in some of these allelochemicals can differentially impact the development of herbivores of varying dietary specialization [13,54,55]. Cassava specialists such as *P*. *manihoti* are physiologically adapted to their host’s chemistry and neutralize cyanogenic compounds through detoxification, excretion, or sequestration [56]. On un-infected plants, *P*. *manihoti* therefore attains superior population levels relative to generalists such as *Pa*. *marginatus*, and the *P*. *manihoti-*specialist wasp *A*. *lopezi* dominates the parasitoid complex. Phytoplasma infection however, may reverse this balance, create niche opportunities for *Pa*. *marginatus* and enable competitive exclusion of *Pa*. *manihoti* on CWB-infected cassava plants. The “tri-trophic interactions” and ‘slow-growth-high-mortality’ hypotheses infer that CWB phytoplasma infection likely increases plant quality and lowers phytotoxin content to such extent that performance of dietary generalists is enhanced, and natural enemy action against specialists is exacerbated [32,57]. Empirical work can reveal to what degree phytoplasma modulates this delicate interplay between plant nutritional quality, herbivore dietary breadth, and the relative impact of parasitism or predation.

Our work provides initial evidence that CWB phytoplasma modifies refuge quality and enemy-free space for two phloem feeders [58], by decreasing host plant suitability for specialists, and facilitating colonization by a generalist phloem feeder. Refuges can confer spatial, temporal or chemical protection from parasitoids or predators, and help sustain multi-trophic interactions [59,60]. As equally observed in an aphid x Brassica system [61], uninfected cassava plants possibly provide specialist herbivores, i.e., *P*. *manihoti*, with a chemically-mediated refuge against (generalist) parasitoids. Phytoplasma infection may remove this refuge, leading to more abundant and speciose parasitoid populations, increased parasitism rates and male-biased sex ratios on CWB-affected plants. Moreover, certain parasitoids (e.g., *A*. *papayae*, *Pseudoleptomastix* sp.) almost exclusively forage on phytoplasma-affected plants.

Enhanced nutritional quality, attenuated phytochemical content and an associated reduction of enemy-free space all directly affect parasitoid communities, enhancing species richness [14,15,62,63]. Although the mechanism is often unclear, pathogen co-infection can either reduce or enhance parasitoid success and affect parasitism rates, e.g. by impacting herbivore fitness [8,10,64]. Cases in which pathogen co-infection lowers predator diversity, and simplifies arthropod food webs have also been reported [12,65]. In our study, CWB-infection increased overall mealybug abundance and diversity at a plant and field-level, lowered parasitism rates on un-infected plants, and sustained species-rich parasitoid complexes on infected plants. Although CWB infection did increase parasitoid richness and diversity at both a plant and field level, this was not reflected in heightened parasitism rates [66]. CWB-infection did not compromise the diversity x function relationship, either in terms of parasitism rates or mealybug host suppression (see Fig 4).

In the case of insect-vectored pathogens such as phytoplasma, parasitoids can act as selection forces in plant-pathogen evolution [6,67]. Parasitic wasps can upset the competitive balance between (colonizing) herbivores and enable apparent competition, as exemplified in a classic study by Settle et al. [68]. Also, hyperparasitoids can directly interfere with establishment and reproductive success of introduced parasitoids, such as *A*. *lopezi* and *A*. *papayae* [69]. These organisms can either release invasive species, such as *P*. *manihoti*, from biological control, or promote population suppression through stabilization of host-parasitoid dynamics [70,71]. It is likely that differential hyperparasitism (for parasitoids) and parasitism (for mealybug hosts) act within our study, but population-level outcomes under field conditions are particularly difficult to interpret or predict, even more so when co-infection occurs with a phytopathogen [72,73].

Aside from causing extensive leaf proliferation and disrupting a plant’s phloem flow, our work reveals how a non-native *Candidatus* phytoplasma infection modifies host quality for sap-feeding homopterans, and generates niche opportunities for higher trophic orders, such as parasitoids or hyperparasitoids. In this process, new habitat is created for multiple species, herbivore population dynamics are altered, and entire (agro-)ecosystems are restructured. CWB phytoplasma affects the success of biological control against both *P*. *manihoti* and *Pa*. *marginatus*, and may determine broader food web function and stability [25,39]. Through its far-reaching impact on species richness, invader success, and food web complexity, CWB phytoplasma might thus take on the role of an ecosystem engineer [49,74].

Our work leaves little doubt that CWB phytoplasma assumes prime ecological importance within Asia’s cassava ecosystems, and alters evolutionary trajectories for several species, including major agricultural pests such as *Pa*. *marginatus* or *P*. *manihoti*. Our work has immediate relevance to the fields of community ecology, invasion biology and biological control, and can guide future cassava breeding initiatives to account for multiple, concurrent stressors [75]. Holistic approaches are now required to assess how phytoplasma x mealybug interactions shape local ecological communities [76]. A full integration of research approaches and reliance upon e.g., next-generation sequencing, molecular ecology, chemical ecology or metabolomics toolkits can provide much-needed insights into the underlying trophic, physiological or hormonal processes (e.g., [4,31,42,77,78]). Among the different research priorities, an in-depth assessment of CWB phytoplasma ecology and transmission dynamics should be included. Also, a (multi-trophic) community perspective will be needed to predict dynamics of invasive pests, such as *P*. *manihoti* and *Pa*. *marginatus*, and to visualize ecological repercussions of CWB at broader spatio-temporal scales [67]. Lastly, the socio-economic implications of the above ecological research cannot be overlooked, as cassava remains one of the world’s prime staple, feed and bio-energy crops, and an immediate source of cash income and livelihood security for millions of Asian resource-poor farmers.

## Supporting information

S1 Table Original supporting dataset for the manuscript. A supporting dataset for the manuscript, with per-plant mealybug, parasitoid and hyperparasitoid abundance records is held in a public data repository, accessible at the following URL: http://dx.doi.org/10.7910/DVN/OERIEZ.
(XLSX)
